# Quercetin Abates Aluminum Trioxide Nanoparticles and Lead Acetate Induced Altered Sperm Quality, Testicular Oxidative Damage, and Sexual Hormones Disruption in Male Rats

**DOI:** 10.3390/antiox11112133

**Published:** 2022-10-28

**Authors:** Amany Behairy, Mohamed M. Hashem, Khaled Abo-El-Sooud, Abeer E. El-Metwally, Bayan A. Hassan, Yasmina M. Abd-Elhakim

**Affiliations:** 1 Department of Physiology, Faculty of Veterinary Medicine, Zagazig University, Zagazig 44519, Egypt; 2 Department of Pharmacology, Faculty of Veterinary Medicine, Cairo University, Giza 12613, Egypt; 3 Pathology Department, Animal Reproduction Research Institute, Giza 3514805, Egypt; 4 Pharmacology Department, Faculty of Pharmacy, Future University, Cairo 11835, Egypt; 5 Department of Forensic Medicine and Toxicology, Faculty of Veterinary Medicine, Zagazig University, Zagazig 44519, Egypt

**Keywords:** aluminum trioxide nanoparticles, lead acetate, quercetin, oxidative stress, inflammation, androgen receptors, tumor necrotic factor alpha, rats

## Abstract

This study examined the effects of exposure to lead acetate (PbAc) and/or aluminum trioxide nanoparticles (Al_2_O_3_NPs) on testicular function. Additionally, the probable reproprotective effects of quercetin (QTN) against Al_2_O_3_NPs and PbAc co-exposure in male Sprague Dawely rats were assessed. Al_2_O_3_NPs (100 mg/kg b.wt.), PbAc (50 mg/kg b.wt.), and QTN (20 mg/kg b.wt.) were orally administered for 60 days. Then, spermiogram, histopathological examinations of the testis and accessory glands, and immunohistochemical detection of androgen receptors (AR) and tumor necrotic factor alpha (TNF-α) were achieved. Moreover, serum levels of male sex hormones and testicular levels of antioxidant indices were estimated. The results showed that Al_2_O_3_NP_s_ and/or PbAc caused significant sperm abnormalities, testicular oxidative stress, and histopathological changes. Furthermore, serum testosterone, LH, and FSH levels significantly decreased, while estradiol levels significantly increased. The Al_2_O_3_NPs and/or PbAc co-exposed group had more obvious disturbances. Furthermore, QTN co-administration significantly reversed the Al_2_O_3_NPs and PbAc-induced testicular histopathological alterations, reduced antioxidant defenses, and altered AR and TNF-α immune expression in testicular tissues. Conclusively, Al_2_O_3_NPs and/or PbAc evoked testicular dysfunction by inducing oxidative injury and inflammation. However, QTN oral dosing effectively mitigated the negative effects of Al_2_O_3_NPs and PbAc by suppressing oxidative stress and inflammation and improving the antioxidant defense system.

## 1. Introduction

In recent years, the usage of engineered nanomaterials for various biomedical and industrial purposes has significantly increased, raising multiple worries concerning harmful consequences on animal and human health [1]. In particular, the toxicological effects of inorganic nanoparticles (NPs) on reproductive organs have lately come to light [2]. Numerous studies have demonstrated that several NPs such as silver (Ag NPs), titanium dioxide (TiO_2_NPs), and aluminum trioxide (Al_2_O_3_NPs) can alter the integrity of the blood–testis barriers (BTB) [3,4,5]. The BTB is a strong structure consisting of tight junctions, gap junctions, and adhesion junctions between adjacent Sertoli cells of the testes close to the base of the seminiferous tubule [6]. It functions as a suitable microenvironment for spermatogenesis and prevents cytotoxic substances from entering the seminiferous tubules, preventing foreign substances’ effect on spermatogenic cells [7]. The underlying mechanisms of NPs crossing BTB remained unclear until the study of Lan and Yang [8], which proposed the “elevator door” hypothesis wherein NPs weakened BTB integrity via induction of inflammatory reactions. Moreover, other studies demonstrated that NPs exert destructive effects on BTB function through disruption of Sertoli cell junctional proteins, oxidative stress, and cell apoptosis [3,9].

Al_2_O_3_NPs are one of the extensively used NPs in food production, agriculture, industry, engineering, medicine, and pharmacy [10,11]. In particular, Al_2_O_3_NPs have proven extremely useful in vaccination biofiltration, drug delivery, and antigen delivery [12]. Al_2_O_3_NPs production in 2010 was around 18,500 tons worldwide. With their steady increase in output, Al_2_O_3_NPs production exceeded 100,000 tons in 2020 [13]. Nevertheless, Al_2_O_3_NPs have been reported to cause the release of reactive oxygen species (ROS), DNA mutations, and pro-inflammatory cytokines, which cause significant damage to the kidneys, brain, liver, and immune system [14]. Recently, in vitro and in vivo studies have revealed that Al_2_O_3_NPs have hepatotoxicity, nephrotoxicity, myocardial toxicity, reproductive toxicity, and neurotoxicity effects [15].

Al_2_O_3_NPs have been reported to penetrate the BTB, induce oxidative stress, generate lipid peroxidation, and injure the testicular biological membranes [5,16,17]. Hamdi [5] reported that Al_2_O_3_-NPs (70 mg/kg b.wt.) oral exposure in albino rats for 28 days induced a substantial reduction in the spermatozoa concentration, impaired sperm morphology, significant increase in DNA damage, histopathological perturbations, and increase in caspase-3 expression intensity. Furthermore, the testicular tissue of Swiss albino male mice orally administered Al_2_O_3_ NPs (15 or 30 mg/kg b.wt. for 5 days) showed high Al retention, increased lipid peroxidation levels, decreased glutathione content, reduced catalase and superoxide dismutase activities, and increased DNA damage [17]. However, little is known about the reprotoxic effect of Al_2_O_3_NPs at long-term exposure and the probable underlying mechanisms.

Different forms of contaminants commonly co-exist in the environment. Heavy metals are widely distributed contaminants that may co-occur with environmental NPs [1]. Heavy metals are among the primary factors reducing human male fertility [18]. Lead acetate (PbAc) is used in various industrial applications, including batteries, smelters, coloring agents, and paints [19]. PbAc has been found in drinking water [20], rice [21], and fruits [22], and humans are thus indirectly absorbed. Absorption of PbAc occurs in the duodenum, where it subsequently binds to proteins in erythrocytes and is transported to distant soft tissues [23]. Small amounts are conjugated in the liver and passed through the urine, while the rest accumulates in tissues, where it damages macromolecules and eventually kills cells [24].

Oxidative stress is considered the primary contributory agent in the pathogenesis of PbAc poisoning [25]. It is classified as a multi-organ toxin as it interferes with many organ functions, including neurological, behavioral, immunological, renal, hepatic, reproductive, ocular, and hematological [26,27,28,29]. PbAc exposure has been associated with decreased sperm concentration, count, and motility and decreased spermatozoa activity [30,31]. Moreover, male mice given increasing levels of PbAc (0.5, 1.0, and 1.5 g/L) in drinking water for 60 days showed a dose-dependent increase in testicular Pb content, reduced sperm quality, and altered histological alterations [32]. Abbaszadeh et al. [33] demonstrated significant decreases in the levels of tissue antioxidants and increases in inflammatory cytokines in the testis of rats orally received 1000 mg of Pb/L drinking water for four weeks. Hence, PbAc could cause reproductive harm via the following mechanisms: oxidative stress, a reduction in testicular antioxidants, toxic effects on spermatocytes, and changes in the hypothalamic–pituitary–gonadal (HPG) axis [30]. Nevertheless, more investigations are still required on the other probable-underlying mechanisms of PB-induced reprotoxic effects, particularly at co-exposure with other pollutants.

In general, the interactions between oxidative stress and inflammation are complicated because one could be a cause and the other a result [34]. In this respect, NPs can generate ROS directly on their surfaces or via macrophage activation, increasing oxidative types, antioxidant production, and inflammation [35]. TNF-α, a major pro-inflammatory cytokine, was overexpressed in many NPs-induced testicular inflammations such as Al_2_O_3_NPs, silica NPs, and zinc oxide NPs [16,36,37]. Previous research suggested that PbAc exposure activates the nuclear factor kappa-light-chain-enhancer of activated B cells (NF-κB) and increases the inhibitory phosphorylation of adenosine monophosphate-activated protein kinase, which facilitates extremely ROS release, as well as the inflammatory cytokines’ upregulation in the testis [31]. Additionally, Basalamah, et al. [38] demonstrated an increase in testicular TNF-α following PbAc exposure (1000 mg/L drinking water for four weeks) in adult male Wistar rats.

Testosterone, a primary androgen of males, is synthesized in Leydig cells under the regulation of luteinizing hormone (LH) released from the anterior pituitary gland [39]. Testosterone is selectively attached to the androgen receptor (AR) in Sertoli cells, and receptor activation triggers the spermatogenic process, sustains the formation of connections between Sertoli cells that make up the BTB [40], and prevents the apoptosis of germ cells [41]. Some NP has been reported to reduce androgen receptor (AR) concomitantly with reduced testosterone level such as Ag NPs [36]. Additionally, AR expression was significantly reduced in the testicular tissue of male Wistar rats orally given PbAc (10 mg/kg b.wt. for 35 days [42].

In this era, there is a global demand to find natural supplements with antioxidant properties that could mitigate the harmful effects of environmental contaminants on man and animal fertility [43,44]. Quercetin (QTN) (3,5,7,30,40-pentahydroxyflavone) is an intriguing polyphenolic flavonoid present in broccoli, tea, red wine, red grapes, cherries, kales, apples, and berries in sufficient amounts. It has been shown to protect against various toxins and diseases [45,46]. The anti-inflammatory, antioxidant, and metal-chelating activities of QTN contribute to its health-promoting benefits [45]. Earlier research highlighted QTN’s protective role against oxidative and inflammatory injuries in tissues such as the kidney, testis, and liver [45,47]. QTN treatment counteracted the cadmium-induced decline of seminiferous tubule diameter, testicular oxidative stress indicators (glutathione peroxidase (GP×), superoxide dismutase (SOD), and malondialdehyde (MDA)), and serum testosterone [48]. QTN has been shown to restore testosterone levels and testicular function by blocking an enzyme responsible for converting testosterone into testosterone glucuronide [49].

Due to their strong affinity to Pb^2+^, Al_2_O_3_NPs products commonly remove Pb from drinking water [50,51,52]. These applications could cause high environmental releases of Al_2_O_3_NPs and Pb mixture. Moreover, the wide industrial and agricultural applications of both Pb [53] and Al_2_O_3_NPs [54] could result in their co-existence in the wastes and their dispersal in the surrounding environment. Moreover, Al_2_O_3_NPs have been reported to have low degradability and can occur in the environment in high concentrations [55]. Additionally, humans could expose to Al_2_O_3_ NPs and/or Pb at levels that highly exceed the national standard through multiple occupational as well as non-occupational sources of exposure. For instance, it has been reported that the maximal level of Pb dust in the workshop in China is 5000 times higher than the national standard [56]. However, the probable effects of Al_2_O_3_NPs and Pb co-exposure on living organisms have been little investigated [57].

As a result, the current study was planned to examine the single and combined effects of Al_2_O_3_NPs and PbAc on sperm parameters, hormonal balance, antioxidant status, androgen receptors (AR) and tumor necrosis factor-alpha (TNF-α) immune expression, and testicular histopathology. Furthermore, to the best of our knowledge, there is no report on the beneficial effects of QTN in conditions where humans or animals are co-exposed to Al_2_O_3_NPs and PbAc, as well as the associated male reproductive disorders.

## 2. Materials and Methods

### 2.1. Chemicals and Reagents

Al_2_O_3_NPs powder (CAS no. AO 301, 101.96 g/mol molecular weight, <100 nm particle size, and purity ≥ 99.5%) was obtained from Alpha Chemika, Cairo, Egypt. Pb Ac (99% purity with molecular weight 379.33) was purchased from Alpha Chemika, Cairo, Egypt. QTN (CAS no. QS 355 and 338.27 g/mol molecular weight) was purchased from Alpha Chemika, Cairo, Egypt.

### 2.2. Animals and Experimental Design

Adult male Sprague Dawley rats (*n* = 70) were obtained from the National Research Center’s breeding unit (Giza, Egypt). All rats were housed in well-ventilated, clean steel mesh cages with a 12-h light-dark cycle at 21–24 °C and 50–60% relative humidity. To keep cages dry, wood-shaving bedding was used. Throughout the experiment, rats had unlimited access to standard rodent chow and tap water. Before testing, rats were acclimated to the experimental circumstances for two weeks.

Rats were weighed and randomly assigned to 7 groups (*n* = 10). G1: Control group: administered distilled water during the trial period. G2: Corn oil group: orally given corn oil (2 mL/kg b.wt./day) [58]. G3: QTN group: orally administered 20 mg QTN/kg b.wt. [59] dissolved in 2 mL/kg. b.wt. corn oil. G4: Al_2_O_3_NPs group: orally administered 100 mg Al_2_O_3_NPs/kg b.wt. dissolved in distilled water. G5: PbAc group: orally administered Pb Ac at a dose of 50 mg/kg b.wt./day dissolved in distilled water. G6: Al_2_O_3_NPs + PbAc group: co-administered Al_2_O_3_NPs and PbAc by the same previous doses. G7: Al_2_O_3_NPs+ PbAc + QTN group: co-administered Al_2_O_3_NPs, PbAc, and QTN at the same previous doses. All treatments were given orally daily between 8 a.m. and 10 a.m. for 60 days 16 gauge). The trial lasted for 60 days to encompass the duration of spermatogenesis in rats, ranging from 56–60 days [60].

Every week, all treatments were adjusted according to the rat’s body weight changes. Throughout the experiment, the rats’ levels of mucous membrane color, discomfort, pain, injury, abnormal behavior, distress, morbidity, respiratory patterns, and mortality were meticulously tracked. Weekly food intake and body change measurements were taken.

### 2.3. PbAc, Al_2_O_3_NPs, and QTN Dose Selection

The PbAc dose was selected from several previous studies that reported that this dose causes male reproductive impairment [61,62,63]. However, the effect of the tested PbAc dose at co-exposure with Al_2_O_3_NPs has not yet been assessed. Additionally, in this study, the oral administration of Pb at a dose of 50 mg/kg b.wt. was used to reveal environmental mimicked Pb exposure. Oral exposure is the common direct route of environmental Pb exposure [64]. Furthermore, Pb exposure resulted in several pathological conditions in the populations exposed to it, even at a low level [65,66]. Thus, regardless of the higher amount of Pb exposure, the cumulative dose of Pb and the individual’s susceptibility are strongly associated with health concerns [67,68].

Due to the limited amount of literature on the toxicity of Al_2_O_3_NPs, particularly reproductive toxicity [69], the selected dose of 100 mg/kg b.wt. Al_2_O_3_NPs have been reported to induce oxidative stress, inflammation, apoptosis, and DNA damage in various organs, including the liver, kidney, and brain [57,70,71]. However, the impact of Al_2_O_3_NPs on testis at the earlier dose has not yet been investigated.

The QTN dose (20 mg/kg b.wt.) has been reported to be efficient in mitigating male reproductive toxicity from exposure to various environmental pollutants and drugs [72,73,74].

### 2.4. Blood Sampling Collection

At the end of the trial (day 60), the rats were fasted for 12 to reduce variability in investigatory parameters, particularly body weight change. Then, rats were weighed and anesthetized with an intramuscular injection of a mixture of ketamine hydrochloride (50 mg/kg b.wt.) and xylazine (5 mg/kg b.wt.). Blood was drawn from each rat from the retro-orbital venous plexus by a well-sterilized glass capillary tube. The blood sample was collected into a centrifuge tube without K_2_EDTA for serum collection for hormonal analyses. All rats were decapitated, the testes were quickly dissected, connective tissues and fat were removed, and weighed.

### 2.5. Testicular Tissues Weights and Sampling

The absolute weight of the testicles was determined using a sensitive weighing balance (Radwag, Model AS220/C/2, Clarkson Laboratory and Supply Inc., Chula Vista, CA, USA). While the following formula determined the relative testicular weight: Relative testis weight = Testis weight/Body weight × 100. Each rat’s left testis was used to measure AL and Pb content. Parts of the testes were rinsed with sterile saline and fixed in 10% buffered formalin for histopathological and immunohistochemical studies of AR and TNF-α. Right testicular samples (0.5 g) were homogenized in 5 mL phosphate buffer (pH 7.4) using an electrical homogenizer and then stored on ice. Supernatants from testicular homogenates were separated using a centrifuge set at 1200× *g* for 20 min at 4 °C, then frozen at −80 °C for later use in measuring antioxidant and oxidative stress biomarkers.

### 2.6. Semen Assessment

The cauda epididymidis from one testis was excised and cut with a sterilized scissor in a petri dish where the spermatozoa were dispersed in 2 mL prewarmed physiological saline solution at 37 °C to assess individual sperm motility [75]. A drop of the epididymal suspension was transferred into a clean glass slide pre-heated at 37 °C and covered with a pre-heated glass cover slide at 37 °C before being examined under a light microscope at high power (400×) magnification. Numerous microscopical fields were investigated to analysis about 200 sperms in these microscopic fields within 2–4 min after their extraction from the epididymis. A subjective scoring between 0 and 100 percent was used to assess the motile sperm cell percentage [76]. A hemocytometer was used to determine the total number of spermatozoa/mL [77]. Each rat’s epididymal fluid was then mixed with the same volume of eosin–nigrosin stain. It spread onto slides that had been prepared to remove grease and debris to identify the morphological abnormalities of sperms under the microscope at a magnification of ×1000 [78]. The results were expressed as percentages.

### 2.7. Analysis of Al and Pb Residues

The liver samples were digested in the microwaves with 1 mL of 30% hydrogen peroxide and 8 mL of nitric acid. Then, the contents of Al and Pb were determined by an inductively coupled plasma–Optical Emission Spectrometer (ICP-OES, model 5100, Agilent, Santa Clara, CA, USA) with Synchronous Vertical Dual View (SVDV). Each measurement series’ intensity was calibrated using a blank and at least three Merck Company standards (Darmstadt, Germany). External reference standards from Merck and standard reference material for trace elements in a quality control sample from the National Institute of Standards and Technology (NIST) were used to verify the instrument readings and ensure the accuracy and precision of the metal measurements.

### 2.8. Hormones Measurements

Serum samples were analyzed for hormone concentrations using enzyme-linked immunosorbent assay (ELISA) kits specific to rats, per the manufacturer’s instructions. Cusabio Biotech Company provided rat testosterone and estradiol assay kits (Wuhan, China). Kamiya Biomedical Company provided rat follicle-stimulating hormone (FSH) and luteinizing hormone (LH) ELISA kits (Seattle, WA, USA) according to Zirkin and Chen [79]’ method.

### 2.9. Oxidative Stress Biomarkers Analysis

Antioxidant enzyme activities and lipid peroxidation level (malondialdehyde (MDA) activity) were determined in testicular tissue homogenate. Commercial ELISA kits (Cusabio Biotech Co., Ltd., Wuhan, China) were used to measure glutathione peroxidase-like activity (GPx) because of the probable measurement of peroxiredoxin enzymatic activity and superoxide dismutase (SOD) [80,81]. The colorimetric assay of Ohkawa et al. [82] was used to determine MDA concentration.

### 2.10. Histopathological Evaluation

Tissue specimens from the right testis, prostate gland, and seminal vesicle were collected from all experimental groups and prepared according to Suvarna et al. [83]. They were fixed, dehydrated with ascending concentrations of ethyl alcohol, cleared in xylene, embedded, and blocked in paraffin. The sections were stained with Hematoxylin and Eosin (H&E) before being examined randomly with a light microscope.

### 2.11. Immunohistochemistry of AR and TNF-α

Testicular tissue sections were dewaxed, rehydrated, and autoclaved in 10 Mm citrate buffer for 10 min at 120°C, as described by Banchroft et al. [84]. Washing with PBS then 0.3% H_2_O_2_ in methanol was applied for 15 m to block endogenous peroxidase. The slides were then blocked by adding a blocking buffer and incubated for 30 min at room temperature after being washed in PBS. According to the avidin–biotin–peroxidase complex protocol of Hsu et al. [85], the primary AR (rabbit monoclonal anti-androgen receptor antibody [ER179(2)]-ChIP Grade (ab108341) primary antibody, Abcam, USA) and TNF-α (Mouse anti-TNF-α antibody-(SC-52746) Santa Crus Biotechnology, Inc., Dallas, TX, USA) antibody were diluted by PBS and incubated/30 m, then washed with PBS three times/3 m each. The slides were treated with a biotinylated polyvalent secondary antibody and then co-incubated for 30 min, followed by washing three times/3 m with buffer. Metal Enhanced (3,3’-Diaminobenzidine) DAB substrate (Abcam, Boston, MA, USA) working solution was applied to the tissue, incubated for 10 min, and then washed off twice/three minutes later with buffer to visualize the reaction.

### 2.12. Statistical Analysis

By SPSS version 14 (SPSS, Chicago, IL, USA), the data were displayed as mean ± standard error (SE). To evaluate the differences between the groups, we ran a one-way analysis of variance (ANOVA) and then used Tukey’s Post-Hoc test for post hoc pairwise comparisons. When the *p* value is <0.05, the results show statistical significance. The computer program Graphpad (ISI Software, Philadelphia, PA, USA) was used.

## 3. Results

### 3.1. Effects on Body WEIGHT Changes and Testicular Weight

Table 1 showed no significant differences in the initial body weights among experimental groups. QTN administration alone revealed a significant (*p <* 0.05) increase (9.04%) in final body weight with a non-significant change in absolute and relative testicular weight relative to the control group.

The individual administration of Al_2_O_3_NP_s_ or PbAc showed a significant (*p* < 0.05) decrease in final body weight by 7.58% and 19.28%, respectively, compared to the control group. Additionally, the PbAc-exposed group showed a significant decline in body weight gain by 75.24% relative to the control group. Moreover, the Al_2_O_3_NP_s_ + PbAc group showed a significant (*p* < 0.05) reduction in final body weight and weight gain by 17.02% and 71.36%, respectively, compared to the control group. In contrast, the reduction in body weight and weight gain change was significantly (*p* < 0.05) minimized in Al_2_O_3_NP_s_+ PbAc + QTN to be 7.98% and 18.44% lower than the control group (Table 1).

The absolute and relative testicular weights were significantly (*p* < 0.05) reduced in the Al_2_O_3_NP_s_ group (31.03% and 25.61%, respectively) and the PbAc group (40.89% and 26.83%, respectively). Of note, the highest reduction in absolute and relative testicular weights was recorded in Al_2_O_3_NP_s_ + PbAc group to be 45.81% and 36.59%, respectively, lower than the control group. On the contrary, co-treatment with QTN in the Al_2_O_3_NP_s_ + PbAc + QTN group improved the reduction in absolute and relative testicular weights to 5.91% and 2.44%, respectively, lower than the control group (Table 1).

### 3.2. Effects on Semen Quality

**Figure 1:**
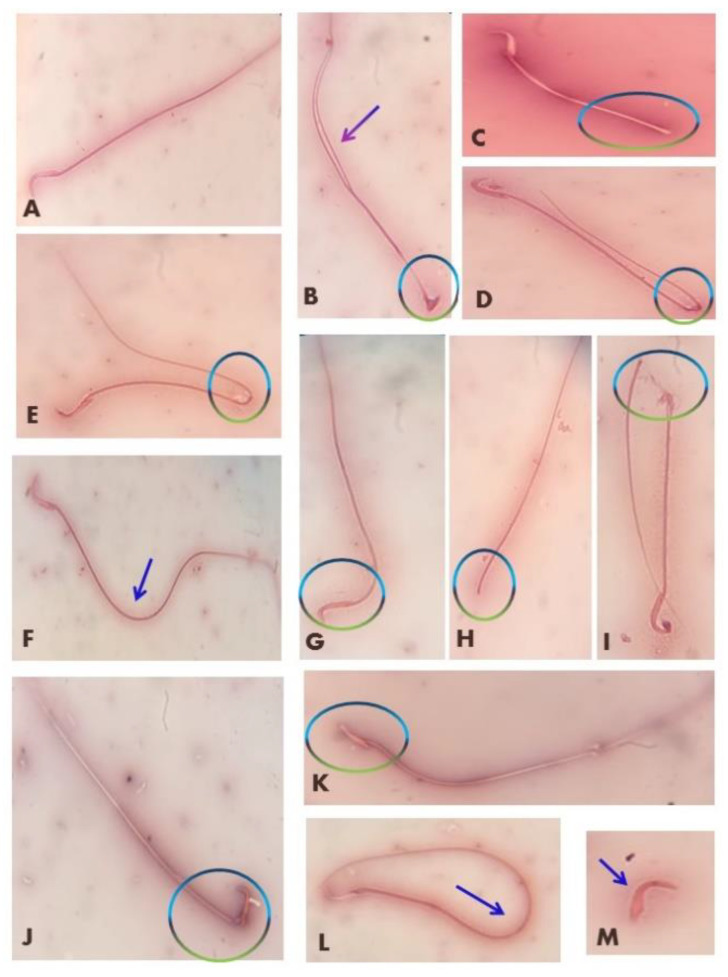
Abnormal spermatozoa induced in rats exposed to Al_2_O_3_NPs and/or PbAc. (**A**) Normal sperm, (**B**) amorphous head with double tail, (**C**) short tail, (**D**,**E**) bent tail, (**F**) curved tail, (**G**) bent head, (**H**) detached head, (**I**) broken tail, (**J**) broken head, (**K**) flattened head, (**L**) looped tail, (**M**) detached tail.

As demonstrated in Table 1, rats orally given QTN displayed a significant increase (*p* < 0.05) in sperm counts (9.33%) and sperm motility (6.38%) with a non-significant change in the percent of sperm abnormalities when compared to the control group. In comparison with the control groups, Al_2_O_3_NP_s_, PbAc, and Al_2_O_3_NP_s_ + PbAc groups showed a significant reduction (*p* < 0.05) in the percentage of motile spermatozoa (43.61%, 62.41%, 81.20%, respectively) and sperm cell counts (17.77%, 27.56%, 46.27%, respectively) compared to control groups. The most reduction was observed in Al_2_O_3_NP_s_ + PbAc co-exposed group when compared with individual exposure to Al_2_O_3_NPs or PbAc. A significant (*p* < 0.05) increase in abnormal sperm percentage was observed when Al_2_O_3_NP_s_ (143.1%) or PbAc (160.08%) were administered alone and in combination (200.12%) compared to the control group. The combined Al_2_O_3_NP_s_ + PbAc group revealed a greater elevation in abnormal sperm percentage when compared to values of both Al_2_O_3_NP_s_ and PbAc groups (*p* < 0.05) (Table 1 and Figure 1).

On the other hand, co-administration of QTN with Al_2_O_3_NP_s_ and PbAc significantly (*p* < 0.05) alleviated the decreases in spermatozoa motility (28.58%) and sperm cells concentrations (7.11%) and significantly dropped the abnormal spermatozoa percentage (95.08%) relative to control group.

### 3.3. Effects on Reproductive Hormones

Individual administration of QTN resulted in a significant (*p* < 0.05) decline in estradiol hormone (38.09%) and a significant increase in testosterone (68.75%), and FSH (33.25%), compared to the control group (Table 2). Instead, serum testosterone levels reduced significantly (*p* < 0.05) in Al_2_O_3_NP_s_ + PbAc groups to be 93.75% lower than in the control group. Oppositely, there was a significant (*p* < 0.05) increase in serum testosterone concentration in the Al_2_O_3_NP_s_ + PbAc + QTN group, compared to the Al_2_O_3_NP_s_+ PbAc groups to the level non-significant from the control group.

There was a decreased but non-significant serum FSH concentration in the rats individually exposed to Al_2_O_3_NP_s_ (27.30%) or PbAc (27.30%). In comparison, the reduction in FSH level was significant (*p* < 0.05) (50.37%) in Al_2_O_3_NP_s_ and PbAc co-exposed rats compared to the control ones. Nevertheless, the QTN co-administration in PbAc and Al_2_O_3_NP_s_ co-exposed rats significantly (*p* < 0.05) minimized the reduction in FSH concentration to 19.85% lower than the control values (Table 2).

Serum LH concentration significantly (*p* < 0.05) decreased in PbAc (46.8%) and Al_2_O_3_NP_s_ + PbAc (52%) groups compared to the control group. Of note, the maximum significant (*p* < 0.05) reduction was observed in serum LH hormone level in the group administered Al_2_O_3_NP_s_ + PbAc together. On the contrary, there was a significant (*p* < 0.05) increase in serum LH concentration in the Al_2_O_3_NP_s_ + PbAc + QTN group compared to the Al_2_O_3_NP_s_ + PbAc co-exposed group and restored to a non-significant level compared to the control value (Table 2).

Table 2 displayed a significant (*p* < 0.05) increase in serum estradiol concentration in Al_2_O_3_NP_s_ (24.37%), PbAc (31.49%), and Al_2_O_3_NP_s_ + PbAc (51.04%) groups, compared to the control group. Oppositely, there was a significant (*p* < 0.05) decrease in serum estradiol concentration in the Al_2_O_3_NP_s_ + PbAc + QTN group compared to the Al_2_O_3_NP_s_ + PbAc group and very nearly to the control group.

### 3.4. Effects on Testicular Oxidative Stress Biomarkers

Regarding SOD, GPx-like activity, and MDA concentrations in testes, there were significant (*p* < 0.05) rises in SOD (65.77%) and GPx-like activity (42.28%) in the QTN group but a significant (*p* < 0.05) decrease (19.48 %) in testicular MDA level as compared to the control group. In comparison with the control groups, Al_2_O_3_NP_s_, PbAc, and Al_2_O_3_NP_s_ + PbAc groups demonstrated a significant (*p* < 0.05) reduction in SOD by 29.70%, 61.19%, and 65.08%, respectively, compared to the control group. Additionally, testicular GPx-like activity level significantly (*p* < 0.05) decreased in the Al_2_O_3_NP_s_ (19.29%), PbAc (20.47%), and Al_2_O_3_NP_s_ + PbAc (44.68%) groups compared to the control group (Table 2). However, both SOD (5.16%) and GPx-like activity (5.52%) concentrations in testicular homogenates significantly (*p* < 0.05) in the Al_2_O_3_NP_s_ + PbAc + QTN group relative to the control group.

Testicular MDA significantly (*p* < 0.05) increased in the Al_2_O_3_NP_s_ (12.61%), PbAc (15.25%), and Al_2_O_3_NP_s_ + PbAc (19.64%) groups compared to the control group (Table 2). Moreover, MDA concentration significantly (*p* < 0.05) decreased (5.58%) in the Al_2_O_3_NP_s_ + PbAc + QTN group compared to the Al_2_O_3_NP_s_ + PbAc groups and restored nearly to the control value.

### 3.5. Changes in Testicular Content of Al and Pb

Al_2_O_3_NP_s_ exposure in Al_2_O_3_NP_s_, PbAc, or Al_2_O_3_NP_s_ + PbAc group showed significant (*p* < 0.05) elevations in testicular Al level in comparison to control groups with the maximum elevation in Al_2_O_3_NP_s_ + PbAc co-exposed group (Table 2). On the contrary, co-administration of QTN for Al_2_O_3_NP_s_ and PbAc co-intoxicated male rats resulted in a significant (*p* < 0.05) reduction in testicular Al residues compared with Al_2_O_3_NP_s_ and PbAc co-exposed rats. Concerning testicular Pb residues, rats exposed to PbAc and/or Al_2_O_3_NP_s_ for 60 consecutive days showed a significant (*p* < 0.05) accumulation in Pb relative to the control groups (Table 2). Moreover, Pb residue significantly increased in Al_2_O_3_NP_s_ + PbAc co-exposed rats compared to the group individually administered PbAc. Oppositely, the co-administration of QTN for PbAc and Al_2_O_3_NP_s_ co-intoxicated male rats revealed a significant (*p* < 0.05) decrease in testicular Pb residue compared with rats co-administered PbAc and Al_2_O_3_NP_s_.

### 3.6. Histopathological Findings

#### 3.6.1. Testis

**Figure 2:**
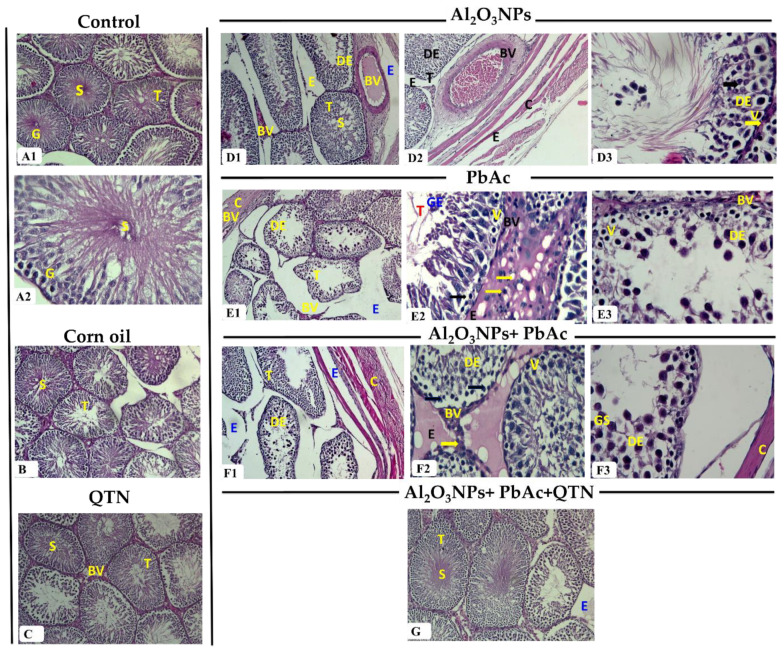
(**A1**) Testis of control group showing normal structure. It consisted of seminiferous tubules (T) lined by stratified germinal epithelium (G) and lumen filled with spermatozoa (S) (H&E, ×100). (**A2**) Testis of the control group showing stratified germinal epithelium (G) lined seminiferous tubules (T) and spermatozoa (S) in the tubular lumen (H&E, ×400). (**B**) Testis of corn oil-treated group showing normal seminiferous tubules (T) lined by several layers of germinal epithelium (G) and spermatozoa (S) in the lumen (H&E, ×100). (**C**) Testis of QTN-treated group showing several rows of germinal epithelium (G) lined seminiferous tubules (T) and spermatozoa (S) in the lumen (H&E, ×100). (**D1**) Testis of Al_2_O_3_NPs-exposed group showing atrophied seminiferous tubules (T) with few spermatozoa (S) in the lumen, disorganized epithelium (DE), severe congestion of the interstitial and sub-capsular blood vessels (BV) and edema (E) (H&E, ×100). (**D2**) Testis of Al_2_O_3_NPs-exposed group showing disorganized epithelium (DE) in the seminiferous tubules (T), interstitial and capsular edema (E), severe congestion of the thickened wall sub-capsular blood vessel (BV) as well as a thick capsule (C) (H&E, ×100). (**D3**) Testis of Al_2_O_3_NPs-exposed group showing epithelial disorganization (DE), sloughing of germinal epithelium, necrotic epithelium, and darkly stained pyknotic nuclei (**Black arrow**), the vacuolated epithelium (V), thickened basement membrane (**Yellow arrow**) and few spermatozoa (S) in lumen (H&E, ×400). (**E1**) Testis of PbAc-exposed group showing atrophied seminiferous tubules (T) with a corrugated outline, sloughed germinal epithelium, the disorganized epithelium (DE), severe congestion of interstitial and sub-capsular blood vessels (BV), edema (E), and thickened capsule (C) (H&E, ×100). (**E2**) Testis of PbAc-exposed group showing sloughed germinal epithelium (GE) within seminiferous tubules (T), necrotic with darkly stained pyknotic nuclei (**Arrow**) and vacuolated (V) germinal epithelium, congestion of interstitial blood vessels (BV), edema (E), and interstitial inflammatory cell infiltrations (yellow arrow) (H&E, ×400). (**E3**) Testis of PbAc-exposed group showing disorganized (DE), sloughed, necrotic and vacuolated (V) germinal epithelium, and congested blood vessel (BV) (H&E, ×400). (**F1**) Testis of Al_2_O_3_NPs and PbAc co-exposed group showing shrunken tubules with different shapes and irregular outlines, germinal disorganization (DE), loss of the germinal epithelium, edema (E), and thick capsule (C) (H&E, ×100). (**F2**) Testis of Al_2_O_3_NPs and PbAc co-exposed group showing germinal disorganization (DE), necrotic with darkly stained pyknotic nuclei (**Arrow**), cellular vacuolation (V), congestion of interstitial blood vessels (BV), edema (E), and interstitial inflammatory cell infiltrations (yellow arrow) (H&E, ×400). (**F3**) Testis of Al_2_O_3_NPs and PbAc co-exposed group showing epithelial disorganization (DE), cellular vacuolation (V), giant spermatids (GS), subcapsular edema (E), and thick capsule (C) (H&E, ×400). (**G**) Testis of Al_2_O_3_NPs and PbAc co-exposure and QTN-treated group showing stratified germinal epithelium-lined seminiferous tubules (T), spermatozoa (S) in the lumen, and mild focal edema (E) (H&E, ×100).

Testis of the control group revealed normal structure. The active mature, functioning seminiferous tubules are lined with complete spermatogenic series and Sertoli cells. Spermatogonia, primary spermatocytes, spermatids, and mature spermatozoa were regularly arranged on the basement membrane. Interstitial spaces were filled with Leydig cells (Figure 2A1,A2). Testis of corn oil and QTN-treated groups were similar to the control group’s structure (Figure 2B,C).

In addition, different pathological changes appeared in tissue sections from Al_2_O_3_NP_s_ and/or PbAc groups. There was spermatogenesis distortion with loss of germinal layers in most tubules. Prominent tubular atrophy, irregular outlines, detachment, and basement membrane thickening were seen. Necrosis with darkly stained pyknotic nuclei, vacuolations, and mild Necrosis of Sertoli cells were noted. Giant spermatids were observed in some tubules. Interstitial edema was noted. Interstitial and sub-capsular blood vessels showed significant congestion, thickening in the wall with vaculations, and vasculitis. Capsule exhibited thickening, edema, and inflammatory cell infiltrations (Figure 2D1–D3,E1–E3). These alterations were increased in severity in Al_2_O_3_NP_s_ with the PbAc-treated group (Figure 2F1–F3). On the other side, Al_2_O_3_NP_s_ + PbAc + QTN-treated group showed apparently normal structure with active spermatogenesis compared with the control group. Few tubules showed mild epithelial vacuolations and edema in some sections (Figure 2G).

#### 3.6.2. Prostate Glands

**Figure 3:**
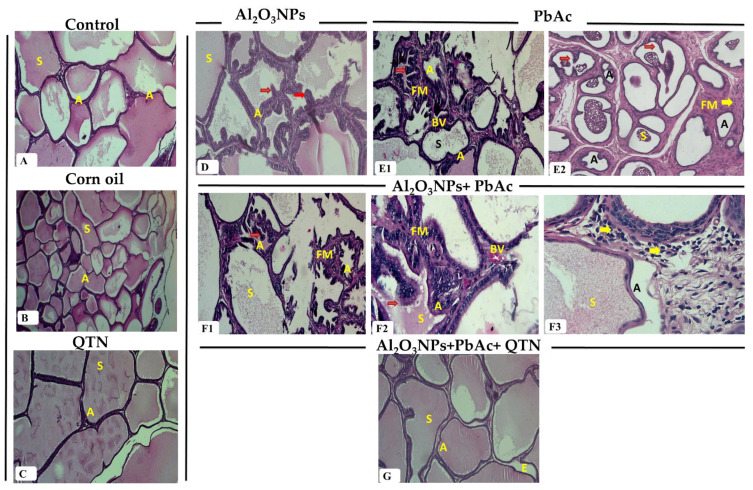
(**A**) Prostate gland of the control group showing normal structure as different sizes and shapes acini (A) with prostatic secretion (S) in the lumen (H&E, ×100). (**B**) Prostate gland of corn oil-treated group showing normal structure with normal acini (A) and secretion (S) in the lumen (H&E, ×40). (**C**) Prostate gland of QTN-treated group showing normal acini (A) and secretion (S) in the lumen (H&E, ×100). (**D**) Prostate gland of Al_2_O_3_NPs-exposed group showing hyperplasia of acini (A) with papillary folds (**Red arrow**) and scanty secretion (S) in the lumen (H&E, ×100). (**E1**) Prostate gland of PbAc-exposed group showing prostatic acinar hyperplasia with papillary folds (**Red arrow**), destructed acini and decreasing acinar size (A), interstitial inflammatory cells and congested blood vessels (BV), thick fibro-muscular stroma (FM) as well as little secretion (S) (H&E, ×100). (**E2**) Prostate gland of PbAc-exposed group showing prostatic acinar hyperplasia with papillary folds (**Red arrow**), destructed acini and decreasing acinar size (A), interstitial inflammatory cells (**Yellow arrow**) and thick fibro-muscular stroma (FM) as well as little secretion (S) (H&E, ×100). (**F1**) Prostate gland of Al_2_O_3_NPs and PbAc co-exposed group showing marked prostatic acinar hyperplasia with papillary folds (**Red arrow**), decreasing acinar size, destructed acini (A), thick fibro-muscular stroma (FM), and little secretion (S) (H&E, ×100). (**F2**) Prostate gland of Al_2_O_3_NPs and PbAc co-exposed group showing acini lined with vacuolated epithelium, acinar hyperplasia with papillary folds (**Red arrow**), destructed acini, thick fibro-muscular stroma (FM), few secretions (S), inflammatory cells around the acini and congested blood vessels (BV) (H&E, ×400). (**F3**) Prostate gland of Al_2_O_3_NPs and PbAc co-exposed group showing prostatic acinar with secretion (S) and inflammatory cells (**Yellow arrow**) around the acini (H&E, ×400). (**G**) Prostate gland of Al_2_O_3_NPs and PbAc co-exposure and QTN-treated group showing normal structure with normal acini (A), secretion (S) in the lumen, and very mild edema (E) (H&E, ×100).

The histological picture of the prostate gland of the control group showed normal aggregations of tubuloalveolar acini with secretion inside their lumen and enclosed within an outer capsule. The acini lined with simple columnar or cuboidal epithelium and embedded in the fibromuscular stroma contained blood vessels (Figure 3A). Prostate glands of corn oil and QTN groups were similar to the control group, as illustrated in Figure 3B,C.

The prostate gland of Al_2_O_3_NP_s_, PbAc, and co-exposure groups exhibited destruction of some acini and decreasing acinar size compared to the control group. Others had vacuolated flattened epithelium with epithelial hyperplasia and marked papillary projections. There were little vacuolated secretions in the lumen. Interstitial tissues showed congested blood vessels, edema, and monocellular inflammatory cells. The fibro-muscular layer was thicker than the control group (Figure 3D,E1,E2,F1–F3). QTN could improve the observed changes caused by Al_2_O_3_NP_s_ and PbAc accompanied by secretion inside the lumen. There were mild papillary projections, edema, and congestion in a few tissue sections (Figure 3G).

#### 3.6.3. Seminal Vesicles

**Figure 4:**
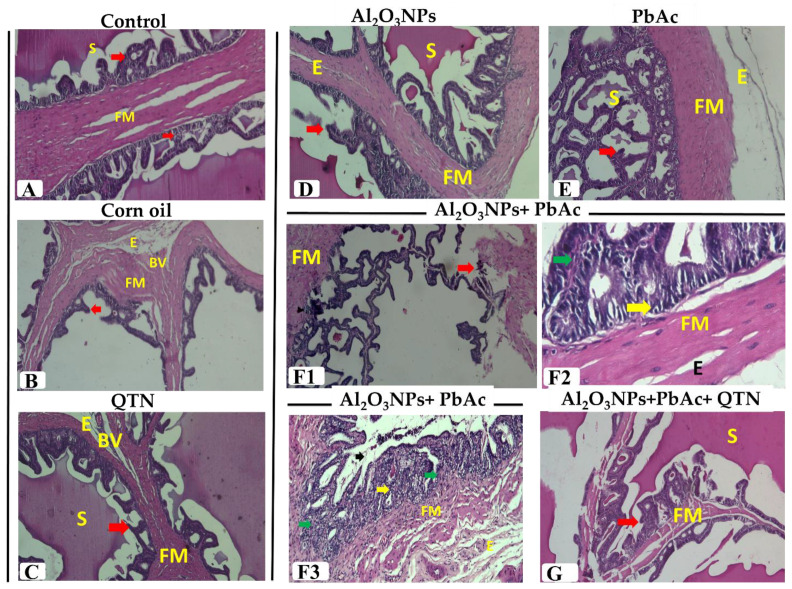
(**A**) Seminal vesicle of control group showing normal branched mucosal folds (**Red arrow**) surrounded by fibro-muscular layer (FM) and secretion (S) in the lumen (H&E, ×100). (**B**) Seminal vesicle of corn oil-treated group showing mucosal folds (**Red arrow**) as well as edema (E) and congestion in the blood vessel (BV) in fibro-muscular layer (FM) (H&E, ×100). (**C**) Seminal vesicle of QTN-treated group showing normal structure as mucosal folds (**Red arrow**) and secretion (S) in the lumen as well as edema (E) and congested blood vessel (BV) in fibro-muscular layer (FM) (H&E, ×100). (**D**) Seminal vesicle of Al_2_O_3_NPs-exposed group showing hyperplastic epithelial folds (**Red arrow**), little secretion (S) in the lumen and thickened fibro-muscular layer (FM) with edema (E) (H&E, ×100). (**E**) Seminal vesicle of PbAc-exposed group showing hyperplastic epithelial folds (**Red arrow**), few secretions (S), subcapsular edema (E), and thickened fibro-muscular layer (FM) (H&E, ×100). (**F1**) Seminal vesicle of Al_2_O_3_NPs and PbAc co-exposed group showing destructed folds (**Red arrow**) and thickened fibro-muscular layer (FM) (H&E, ×100). (**F2**) Seminal vesicle of Al_2_O_3_NPs and PbAc co-exposed group showing vacuolated epithelium (**Yellow arrow**), interstitial mononuclear inflammatory cells (**Green arrow**)**,** and thickened fibro-muscular layer (FM) with edema (E) (H&E, ×400). (**F3**) Seminal vesicle of Al_2_O_3_NPs and PbAc co-exposed group showing destructed folds (**arrow**) vacuolated epithelium (**Yellow arrow**), mononuclear inflammatory cells (**Green arrow**)**,** thickened fibro-muscular layer (FM), and edema (E) (H&E, ×100). (**G**) Seminal vesicle of Al_2_O_3_NPs and PbAc co-exposure and QTN-treated group showing normal mucosal folds (**Red arrow**) surrounded by fibro-muscular layer (FM) and secretions (S) in the lumen (H&E, ×100).

As illustrated in Figure 4A–C, seminal vesicles of control, corn oil, and QTN-treated groups showed that the normal branched convoluted mucosal folds surrounded by smooth muscle and the lumen filled with secretion. Pseudostratified columnar epithelium with highly vesicular nuclei and foamy cytoplasm lined the folds. A well-developed fibro-muscular capsule surrounded the gland from which trabeculae passed through them. Mild blood vessels congestion was observed in a few sections of corn oil and QTN-treated groups.

### 3.7. Immunohistochemistry Analysis

**Figure 5:**
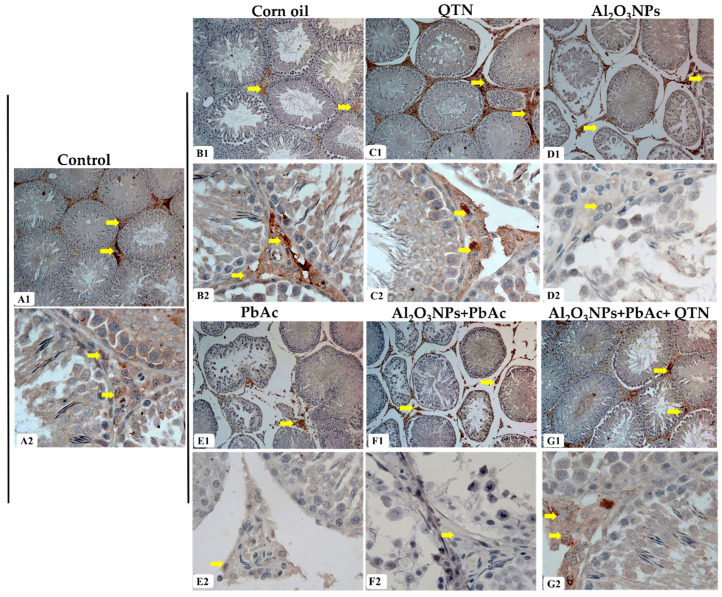
(**A1**,**A2**) Testis of the control group showing strong interstitial cell staining for androgen receptors and lack of immuno-reaction in seminiferous tubules. (**B1**,**B2**) Testis of corn oil-treated group showing normal immuno-expression in the interstitial cells. (**C1**,**C2**) Testis of QTN-treated group showing normal expression of immuno-staining of interstitial cells. (**D1**,**D2**) Testis of Al_2_O_3_NPs-exposed group showing moderate immuno-expression of the androgen receptor. (**E1**,**E2**) Testis of PbAc-exposed group showing weak androgen receptor expression. (**F1**,**F2**) Testis of Al_2_O_3_NPs and PbAc co-exposed group showing weak androgen receptor expression. (**G1**,**G2**) Testis of Al_2_O_3_NPs and PbAc co-exposed and QTN-treated group showing strong androgen receptor expression in the interstitial cells (Androgen receptor immunostain, ×100 for (**A1**–**G1**) and ×400 (**A2**–**G2**)). Yellow arrows denoted AR-positive immune stained cells.

As shown in Figure 5, the testis of control (A1,A2), corn oil (B1,B2), and QTN (C1,C2) rats showed normal AR expression. It stained interstitial cells with brown coloration and a lack of immune reaction in seminiferous tubules. On the contrary, it was observed that there was a noticeable reduction in the AR expression in the testis of Al_2_O_3_NP_s_ and/or PbAc-treated groups (Figure 5D1,D2,E1,E2,F1,F2). In contrast, the AR expression in Al_2_O_3_NP_s_ + PbAc + QTN-treated groups was improved to the extent like that in the testis of the control group (Figure 5G1,G2).

**Figure 6:**
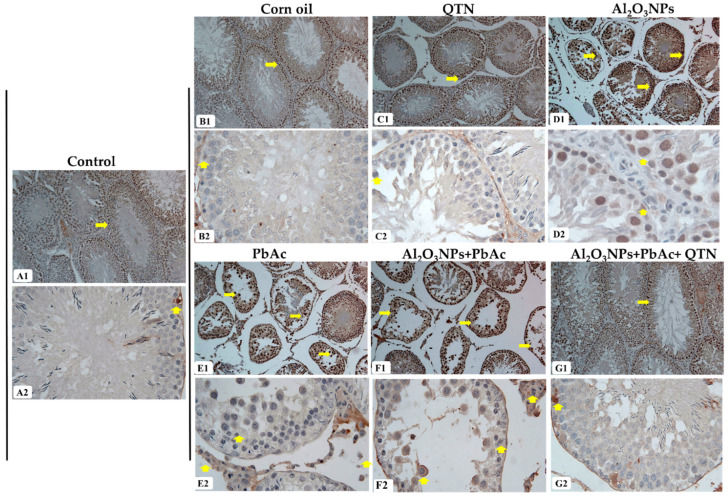
(**A1**,**A2**) Testis of the control rat showing weak TNF-α immuno-stain in the cytoplasm of few spermatogenic cells. (**B1**,**B2**) Testis of corn oil-treated rat showing weak expression for TNF-α. (**C1**,**C2**) Testis of QTN-treated rat showing weak immuno-labeling of some spermatogenic cells. (**D1**,**D2**) Testis of Al_2_O_3_NPs-exposed group showing numerous positive spermatogenic cells and Leydig cells represented by brown color. (**E1**,**E2**) Testis of the PbAc-exposed group showing a strong positive reaction in the cytoplasm of spermatogenic cells and Leydig cells. (**F1**,**F2**) Testis of Al_2_O_3_NPs and PbAc co-exposed group showing strong positive TNF-α immuno-stain (**G1**,**G2**) Testis of Al_2_O_3_NPs and PbAc co-exposed and QTN-treated group showing weak TNF-α positive cells (TNF-α immuno-stained, ×100 for (**A1**–**G1**) and ×400 (**A2**–**G2**)). Yellow arrows denoted TNF-α-positive immune stained cells.

As displayed in Figure 6, the testis of control (A1,A2), corn oil (B1,B2), and QTN (C1,C2)-treated rats showed weak expression for TNF-α. However, TNF-α level expression was markedly increased in Al_2_O_3_NP_s_ (D1,D2), PbAc (E1,E2), and Al_2_O_3_NP_s_ + PbAc (F1,F2) co-exposed rats compared to the control group. There was an obvious decrease in TNF-α expression Al_2_O_3_NP_s_ + PbAc + QTN-treated groups (Figure 6G1,G2).

## 4. Discussion

Body weight changes are considered one of the sensitive indicators of the adverse effects of toxic chemicals [86]. In the current experiment, PbAc-exposed groups (Pb and PbAc + Al_2_O_3_NPs) showed a significant reduction in their body weight compared to the control group. The body weight reduction associated with PbAc exposure could result from Pb-induced reduced food intake, increased catabolic state, and disturbed nutrient metabolism caused by changing zinc-dependent enzymes [87]. On the other hand, the reduction in the body weight gain was not significantly different in the Al_2_O_3_NP_s_-exposed group compared to the control group. Comparably, other NPs have not evoked significant changes in body weight gains, despite other negative effects, even at large doses up to 1 g/kg b.wt. [88,89]. Of note, an obvious restoration of the body weight was recorded in the PbAc + Al_2_O_3_NPs + QTN-treated group. Comparably, QTN reduced the weight loss induced by other NPs such as iron oxide NPs [90]. In this regard, QTN supplementation has been reported to increase the skeletal muscles’ insulin-stimulated glucose uptake and antioxidant capacity, positively reflecting on health and body weight [91]. QTN’s antioxidant capacity has been related to its chemical structure, particularly the presence and position of the catechol group in the B ring and the hydroxyl (-OH) group, which control the redox mechanism and boost free radical scavenging ability [92]. High oxidative stress has been shown to impair mitochondrial production in skeletal muscle [93] and to influence the rate of cellular adenosine triphosphate synthesis [94].

The current study revealed a significant reduction in the testis weight and gonadosomatic index in Al_2_O_3_NP_s_, PbAc, and Al_2_O_3_NP_s_ + PbAc exposed groups. The substantial decrease in the weight of the testis after Al_2_O_3_NP_s_ and/or PbAc exposure was accompanied by alterations in their histology, such as tubular atrophy, detachment, and Sertoli cell necrosis. Comparable reductions in the testis weight and gonadosomatic index have previously been reported following PbAc [95] and Al_2_O_3_NP_s_ [16] exposure in rats. Various reasons might be responsible for the earlier reductions, including spermatogenesis inhibition and reduction in germ, spermatid cells, and testosterone levels [16,96]. On the contrary, the QTN co-treatment significantly restored the testis weight and gonadosomatic index in the Al_2_O_3_NP_s_ + PbAc + QTN group. Consistent with the earlier findings, QTN restored the reduction in testis weight resulting from exposure to other NPs such as TiO_2_NPs [97]. As revealed by the histopathological findings, the QTN-associated reinstatement of the testicular architecture could be responsible for the restoration of the testis weight.

The quality of sperm is the most important predictor of male fertility [98]. In the current study, the exposure to Al_2_O_3_NP_s_ and/or PbAc decreased sperm count and sperm motility while it increased the abnormal sperm count. Comparable impaired sperm parameters were observed in rats exposed to Al_2_O_3_NP_s_ [5] or PbAc [38,99]. The reduction in sperm motility was most likely caused by the influence of Al_2_O_3_NP_s_ on mitochondrial function. In this regard, in an in vitro study using C18-4 cells, Braydich-Stolle et al. [100] reported that Al_2_O_3_NP_s_ could cross the membrane and connect to mitochondria and sperm acrosomes. On the other hand, Leydig cells, which secrete testosterone, are considered the primary target for Pb, which could explain the depleted population of spermatogonia, spermatocytes, and spermatids [101]. Additionally, Pb has been reported to adversely affect the male accessory glands [102], which is apparent in the histopathological findings, with their important role in sperm physiology [103]. Of note, compared to individual exposures, the toxic effects of Al_2_O_3_NP_s_ and PbAc resulted in a more dramatic reduction in sperm count, viability, and increased sperm abnormalities suggesting their synergistic interaction. On the contrary, QTN oral dosing significantly repaired the sperm-impaired parameters resulted from Al_2_O_3_NP_s_ and PbAc co-exposure. Previous studies proposed that the positive effects of QTN on sperm characters could be partly related to its antioxidant activity [104,105]. Additionally, Taepongsorat et al. [106] suggested that QTN might indirectly affect sperm quality via the stimulation of the sex organs (testis and accessory sex organs) at the cellular and organ levels. In line with the earlier suggestion, the histopathological findings revealed the significant restoration of the testis, seminal vesicles, and prostate architecture in Al_2_O_3_NP_s_ + PbAc + QTN-treated rats.

Male sex hormones are key in male fertility as they affect sperm production and health [107]. Herein, the single exposure Al_2_O_3_NP_s_ and PbAc significantly increased the estradiol level and decreased testosterone, FSH, and LH but without significant difference compared to the control group. However, the co-exposure to Al_2_O_3_NP_s_ and PbAc significantly increased serum estradiol while it significantly decreased serum testosterone, FSH, and LH compared to the control group. Comparable disturbance in the male sex hormone balance was earlier reported with exposure to Al_2_O_3_NP_s_ [16] or PbAc [108]. Given this, Lan and Yang [8] reported that NPs exposure could cause inflammation and affect Leydig cells, lowering testosterone serum levels and compromising the BTB. Despite the scarce information on the effect of Al_2_O_3_NPs on the pituitary gland and hypothalamus, other NPs such as Ag NPs [109] and TiO_2_NPs [110] have been reported to alter HPG. Moreover, Pb toxicity in rats has been reported to cause a deformity in the pituitary gland and hypothalamus, thus affecting LH secretion [111,112]. On the contrary, QTN oral dosing in Al_2_O_3_NP_s_ and PbAc co-exposed rats significantly corrected the male hormones balance. These findings could be related to the earlier reported ability of QTN to correct the defects in HPG axis imbalance [113]. Moreover, Sharma et al. [114] reported that the QTN-induced increase in antioxidant defense of Sertoli and Leydig cells is highly associated with increased testosterone, FSH, and LH levels. The same authors proposed that QTN may also protect the pituitary gland and subsequently enhance the level of FSH and LH.

Testicular oxidative stress has been known as a chief feature in male infertility [115]. Herein, Al_2_O_3_NP_s_ or PbAc exposure provoked an obvious testicular oxidative stress in terms of reduced antioxidants (SOD and GPx-like activity) and increased lipid peroxidation (MDA) levels. Moreover, co-exposure to Al_2_O_3_NP_s_ and PbAc resulted in a more dramatic decline in SOD and GPx-like activity and an increase in MDA, indicating a synergistic toxic effect of concurrent exposure rather than exposure alone. Similarly, Yousef et al. [17] verified the depletion of testicular antioxidants, including GPx-like activity, glutathione S-transferases (GST), catalase (CAT), SOD, and reduced glutathione (GSH) in rats that orally received 70 mg Al_2_O_3_NP_s_/kg b.wt. for 75 days. Moreover, Algefare et al. [103] demonstrated that PbAc decreased antioxidant enzyme activity and increased MDA levels in the testes of rats intraperitoneally injected 20 mg PbAc/kg b.wt. for 4 weeks. In this regard, Pb, like most divalent metals, is bound in tissues by ionic or coordination bonds and commonly bound to small peptides, cysteine, methionine, selenomethionine, and enzymes affecting their activity [101]. Also, Pb has been reported to induce free radical damage by two distinct pathways, the release of hydrogen peroxides, and singlet oxygen, reflected in increased MDA levels as the lipid peroxidation end product, and the direct exhaustion of antioxidant reserves [116]. Several studies also verified that Al_2_O_3_ NPs are involved in the ROS generation, thereby leading to a decrease in the antioxidant enzyme activities [117,118]. On the contrary, QTN treatment significantly re-established the depleted antioxidant enzymes and repressed the incremented MDA resulted from Al_2_O_3_NP_s_ and PbAc co-exposure. Several earlier studies confirmed the potent antioxidant effect of QTN [105,119]. In this respect, Tvrdá et al. [120] verified that QTN has a strong scavenging capacity for ROS in favor of the catechol moiety and free hydroxyl groups in its structure, guards sperm from ROS, and retains the male germ cells function. Moreover, QTN has been found to directly eliminate ROS and hydroxyl radicals and renovate endogenous redox homeostasis through increasing glutathione levels and eliminating free radical enzyme systems [121].

Regarding Al accumulation in testicular tissues, the current findings revealed that the control, corn oil, and QTN groups initially had minimal amounts of Al in their testicular tissue. In line with these findings, in the previous study by HM O et al. [122], minimal amounts of Al have been found in various organs, including the control group’s liver, kidney, and spleen. The wide distribution of Al in the environment because of its diverse uses, especially in Al containers, utensils, and water purifiers, could be responsible for its presence in the groups that have not received it as oral dosing [123]. Of note, the PbAc group had significant concentrations of testicular Al compared to the control group. The PbAc exposure had been known to cause renal impairment [124], which might affect the excretion of Al and lead to its accumulation in the tissue. Earlier reports confirmed that impaired renal function considerably increases Al accumulation risk [125,126,127]. In the Al_2_O_3_NP_s_ and Al_2_O_3_NP_s_ + PbAc-exposed rats, significant concentrations of Al were found in their testicular tissues, with the maximum amount in the Al_2_O_3_NP_s_ + PbAc co-exposed ones. In this regard, Al_2_O_3_NP_s_ have the potential to cross intestinal barriers and enter the bloodstream. This transport procedure involves binding blood ligands and delivering them via receptor-mediated phagocytosis, endocytosis, and pinocytosis [128]. Moreover, Al_2_O_3_NPs have been reported to penetrate the BTB and injure the testicular biological membranes [5,17,18]. Additionally, Yousef et al. [11] have reported that the accumulation of Al_2_O_3_NP_s_ in the hippocampus of rats affected mitochondrial membrane function and lipoprotein integrity. Interestingly, a relation between dysfunctions of the brain and testis has been evident in recent studies [129,130]. Of note, the marked accumulation of Al in testicular tissues of the Al_2_O_3_NP_s_ + PbAc-co-exposed group could be related partly to the Pb-induced-impaired excretion of Al [125,126,127]. Additionally, NPs have been known for their large surface area that allows heavy metals to adsorb [131]. The heavy metals can enter the organism as a free ion and/or NPs-heavy metals complex, and NPs can act as carriers for their transport within the organism [132]. The earlier fact could elucidate the presence of minimal Pb in Al_2_O_3_NP_s_-exposed rats and significant Pb concentrations in Al_2_O_3_NP_s_ + PbAc co-exposed rats. Moreover, some earlier reports confirmed that Pb can across BTB [133,134,135].

On the contrary, Pb and AL residues significantly decreased in the testicular tissue of Al_2_O_3_NP_s_ + PbAc + QTN-treated rats compared to those co-exposed to Al_2_O_3_NP_s_ + PbAc. It is proposed that QTN can reduce the Al and Pb concentrations in the testicular tissue by forming complexes. In this regard, it has been reported that the catechol function site in the QTN structure has the highest complexation power toward Pb [136]. Furthermore, QTN has been proposed as a potential metal chelator. The hydroxyl and carbonyl groups in the C ring of QTN are reported to be the chief metal complexing domains interacting with metal [137]. Moreover, QTN has been reported to efficiently protect the BTB integrity [138], which may partly explain its role in reducing Pb and Al accumulation in the testis.

The testis is a sex gland that produces sperm and androgens as part of the HPG axis [139]. Seminal vesicles and prostate glands are important male accessory sex glands that play an important role in sperm motility, capacitation, and survival [140,141]. The present study revealed that the testis, seminal vesicle, and prostate gland of Al_2_O_3_NPs and/or PbAc-exposed rat showed various pathological perturbations. Consistent with these findings, Al_2_O_3_NP and PbAc induced comparable testicular lesions in the studies of Hamdi [5] and Abdrabou et al. [142], respectively. Additionally, PbAc adversely impacted the histological architecture of seminal vesicles and prostate in the earlier studies of Aldaddou et al. [143] and Dhurvey et al. [102]. Moreover, other NPs such as TiO_2_NPs [144] and zinc oxide NPs [122] caused hyperplasia, congestion, and desquamation of the prostate’s epithelial and lining cells, as well as congestion in the seminal vesicle. The earlier effects of Al_2_O_3_NP and PbAc could be highly linked to their oxidative stress-inducing effects. In contrast, the testis, seminal vesicle, and prostate gland histoarchitecture were significantly restored in Al_2_O_3_NP and PbAc co-exposed rats with QTN oral dosing. QTN antioxidant efficacy could be responsible for reestablishment of testis and male accessory gland function and architecture. In addition, QTN therapy decreased the levels of the oxidative stress metabolite F2-isoprostane and prostaglandin E2 in expressed prostatic secretions (EPS) while increasing the levels of prostatic β-endorphins [145,146,147]. Moreover, in a recent study, QTN protected against chronic histologic prostatitis in rats [148].

The present study revealed that the individual and combined administration of Al_2_O_3_NP_s_ and PbAc reduced the immune expression of AR compared to the control group. The reduced testosterone content in the testis of Al_2_O_3_NPs and PbAc-exposed groups could be responsible for their reduced AR immunoexpression because of their positive correlation. Comparably, in silica NP-exposed mice, serum testosterone levels were considerably reduced parallel with decreased mRNA expression of AR and genes regulating testosterone synthesis, resulting in spermatogenesis malfunction [36]. Furthermore, Huang et al. [149] demonstrated that PbAc induced a dose-dependent reduction in AR expression in the spermatogenic cells, Sertoli cells, and Leydig cells. Given this, Pb is a known endocrine disruptor with estrogenic properties, and it may be responsible for antagonizing AR expression in male Wistar rats [42,150,151]. The decrease in testicular AR expression observed in PbAc-exposed rats may also be explained by a reduction in serum testosterone levels [42]. Nevertheless, QTN mitigated AR immune expression in the Al_2_O_3_NP_s_ + PbAc + QTN-treated group, which may be linked to its antioxidant properties. Additionally, treating primary cultures of Leydig cells by QTN resulted in a dose-dependent upregulation of AR [152]. This might be because QTN promotes steroidogenesis in Leydig cells [153]. Moreover, as testosterone is known to regulate AR expression through 5α-reductase, the QTN-induced increase in AR immunoexpression possibly related to the aromatase-inhibiting property of QTN preventing the conversion of androgen into estrogen [154]. Overall, the considerable enhancement of semen quality, hormonal balance, and testicular oxidative status in the QTN-treated groups could be linked to its chelating activity, powerful antioxidant properties, and efficacy in balancing the HPG axis.

Pro-inflammatory cytokines like TNF–α cytokines in the testis may have certain physiological functions. However, when these cytokines are higher than normal, they harm sperm [155]. In the current experiment, the TNF-α level immune expression was significantly increased in the testicular tissue sections of Al_2_O_3_NP_s_, PbAc, and Al_2_O_3_NP_s_ + PbAc-exposed rats compared to the control group. Comparably, El-Khadragy et al. [156] reported an elevation in the proinflammatory cytokine TNF-α in the testis of rats after intra-peritoneal Pb exposure (20 mg/kg b.wt.) for 7 days. Additionally, the pro-inflammatory effects of NPs have been recently documented [157,158] and correlated with the binding of proteins to NP surfaces. For instance, the adsorption of TNF-α on carbon black, TiO_2_NPs, and aluminum oxide hydroxide (AlOOH) NP has been reported [159]. Additionally, exposure of rats to Al_2_O_3_NP_s_ daily for 75 days resulted in a significant elevation of testicular levels of TNF-α [16]. As, the inflammatory reactions within the testis are strongly connected with oxidative stress [160], the Al_2_O_3_NP_s_ and PbAc-induced oxidative stress could be responsible for the increase in TNF–α immunoexpression. On the contrary, QTN co-administration suppressed TNF-α immune expression in the Al_2_O_3_NP_s_ + PbAc + QTN-treated group, which reflects its anti-inflammatory effect [161]. In this regard, the study of Nair et al. [162] showed that a possible mechanism of QTN-mediated suppression of TNF-α expression is mediated in downregulating NF-κβ1 gene expression.

## 5. Conclusions

The results showed negative effects of Al_2_O_3_NPs and PbAc alone or in combination on the testicular function and architecture. Various pathways, including the induction of oxidative stress, lipid peroxidation, disruption of the HPG axis and sex hormone imbalance, spermiogram issues, and histopathological changes, have been implicated in Al_2_O_3_NPs and PbAc reprotoxic effect. Enhancing TNF-α and suppressing AR immune expression in testicular tissue was another possible mechanism. Of note, the combination of Al_2_O_3_NPs and PbAc had greater reproductive toxicity than either one alone. However, further studies are warranted on Al_2_O_3_NPs and PbAc co-exposure outcomes at lower concentrations for longer durations. The current investigation also verified the potential therapeutic benefits of QTN against Al_2_O_3_NPs and PbAc-induced altered reproductive parameters in experimental rats, which were thought to be caused by its anti-oxidative anti-inflammatory properties, and TNF-α and AR modulation.

## Figures and Tables

**Table 1:** Effect of quercetin (QTN) oral dosing on body weight change, gonadosomatic index, and sperm characteristics of adult male Sprague Dawley rats exposed to aluminum trioxide nanoparticles (Al_2_O_3_NPs) and/or lead acetate (Pb Ac) for 60 days.

Estimated Parameters	Experimental Groups						
	Control	CO	QTN	Al_2_O_3_NPs	Pb Ac	Al_2_O_3_NPs + Pb Ac	Al_2_O_3_NPs + Pb Ac + QTN
Initial Body weight (g)	182.00 ± 0.71	181.33 ± 3.79	182.00 ± 2.55	186.67 ± 3.70	185.33 ± 1.89	188.33 ± 3.01	189.33 ± 2.05
Final body weight (g)	250.67 ^b^ ± 10.02	245.00 ^b^ ± 14.71	273.33 ^a^ ± 15.74	231.67 ^bc^ ± 1.65	202.33 ^c^ ± 7.10	208.00 ^c^ ± 8.64	270.67 ^a^ ± 14.27
Body weight change (g)	68.67 ^ab^ ± 10.73	63.00 ^ab^ ± 12.90	86.67 ^a^ ± 16.41	45.00 ^bc^ ± 2.94	17.00 ^c^ ± 6.75	19.67 ^c^ ± 7.55	81.33 ^a^ ± 14.06
Testes weight (g)	2.03 ^a^ ± 0.08	1.92 ^a^ ± 0.07	2.19 ^a^ ± 0.02	1.40 ^b^ ± 0.12	1.20 ^b^ ± 0.11	1.10 ^b^ ± 0.11	2.15 ^a^ ± 0.20
Gonadosomatic index (%)	0.82 ^a^ ± 0.05	0.80 ^a^ ± 0.08	0.81 ^a^ ± 0.05	0.61 ^b^ ± 0.06	0.60 ^b^ ± 0.07	0.52 ^b^ ± 0.03	0.80 ^a^ ± 0.07
Sperm count (sp.cc/mL × 125 × 10^4^)	75.00 ± 3.46 ^bc^	76.00 ^b^ ± 1.53	82.00 ^a^ ± 2.08	61.67 ^d^ ± 1.20	54.33 ^e^ ± 0.88	40.3 ^f^ ± 0.88	69.67 ^c^ ± 1.76
Sperm abnormalities (%)	16.66 ^d^ ± 1.09	17.50 ^d^ ± 0.58	13.16 ^d^ ± 1.59	40.50 ^b^ ± 2.08	43.33 ^ab^ ± 2.40	50.00 ^a^ ± 5.20	32.50 ^c^ ± 1.60
Sperm motility (%)	88.67 ^b^ ± 0.88	85.33 ^b^ ± 0.33	94.33 ^a^ ± 1.20	50.00 ^d^ ± 2.89	33.33 ^e^ ± 1.67	16.67 ^f^ ± 0.88	63.33 ^c^ ± 1.67

Means within the same row carrying different superscripts (a, b, c, d, e, and f) are significantly different at *p* < 0.05. The values shown are means ± SE. *n* = 10.

**Table 2:** Effect of quercetin (QTN) on serum levels of male hormones and oxidative stress indicators and testicular tissue content of lead (Pb) and aluminum (Al) of adult male Sprague Dawley rats exposed to aluminum trioxide nanoparticles (Al_2_O_3_NPs) and/or lead acetate (Pb Ac) for 60 days.

Estimated Parameters	Experimental Groups						
	Control	CO	QTN	Al_2_O_3_NPs	Pb Ac	Al_2_O_3_NPs + Pb Ac	Al_2_O_3_NPs + Pb Ac + QTN
Testosterone (pg/mL)	0.16 ^bc^ ± 0.02	0.18 ^b^ ± 0.00	0.27 ^a^ ± 0.06	0.10 ^bc^ ± 0.00	0.09 ^c^ ± 0.00	0.01 ^d^ ± 0.00	0.15 ^bc^ ± 0.02
Estradiol (mIU/mL)	30.90 ^c^ ± 1.09	31.50 ^c^ ± 0.67	19.13 ^d^ ± 2.02	38.43 ^b^ ± 1.13	40.63 ^b^ ± 3.28	46.67 ^a^ ± 1.64	26.97 ^c^ ± 2.27
LH (mIU/mL)	2.50 ^ab^ ± 0.11	2.53 ^ab^ ± 0.10	3.23 ^a^ ± 0.66	1.87 ^bc^ ± 0.09	1.33 ^c^ ± 0.13	1.20 ^c^ ± 0.11	2.30 ^b^ ± 0.11
FSH (mIU/mL)	4.03 ^bc^ ± 0.47	4.20 ^b^ ± 0.66	5.37 ^a^ ± 0.42	2.93 ^cd^ ± 0.28	2.93 ^cd^ ± 0.06	2.00 ^d^ ± 0.07	3.23 ^bc^ ± 0.06
SOD (IU/g. protein)	44.99 ^b^ ± 1.31	42.42 ^b^ ± 2.33	74.58 ^a^ ± 3.84	31.63 ^c^ ± 1.31	17.46 ^d^ ± 0.48	15.71 ^d^ ± 0.87	42.67 ^b^ ± 1.28
GPx-like activity (IU/g. protein)	66.25 ^b^ ± 1.31	64.37 ^b^ ± 2.64	94.26 ^a^ ± 2.13	53.47 ^c^ ± 2.06	52.69 ^c^ ± 2.25	36.65 ^d^ ± 1.60	62.59 ^b^ ± 3.60
MDA (nmol/g. protein)	113.74 ^d^ ± 0.98	115.08 ^cd^ ± 2.31	91.58 ^e^ ± 0.88	128.08 ^b^ ± 0.91	131.08 ^ab^ ± 2.08	136.08 ^a^ ± 3.35	120.09 ^c^ ± 1.49
Pb residues (ppm)	ND	ND	ND	0.08 ^d^ ± 0.00	60.75 ^b^ ± 0.01	0.93 ^a^ ± 0.0	0.57 ^c^ ± 0.01
Al residues (ppm)	8.13 ^e^ ± 0.76	6.90 ^e^ ± 0.56	5.0 ^e^ ± 0.13	64.05 ^b^ ± 1.11	25.90 ^c^ ± 0.33	138.50 ^a^ ± 2.29	19.60 ^d^ ± 0.33

LH: luteinizing hormone; FSH: follicle-stimulating hormone; SOD: super oxide dismutase; GPx: glutathione peroxidase; MDA: malondialdehyde. Means within the same row carrying different superscripts (a, b, c, d, and e) are significantly different at *p* < 0.05. Values shown are means ± SE. *n* = 10 group.

## Data Availability

All datasets generated for this study are included in the article.
